# Perilla Oil Supplementation Improves Hypertriglyceridemia and Gut Dysbiosis in Diabetic KKAy Mice

**DOI:** 10.1002/mnfr.201800299

**Published:** 2018-11-08

**Authors:** Feng Wang, Hangju Zhu, Mingyuan Hu, Jing Wang, Hui Xia, Xian Yang, Ligang Yang, Guiju Sun

**Affiliations:** ^1^ Key Laboratory of Environmental Medicine and Engineering of Ministry of Education, and Department of Nutrition and Food Hygiene, School of Public Health Southeast University Nanjing China; ^2^ Tianjin Institute of Environmental and Operational Medicine Tianjin China; ^3^ Jiangsu Cancer Hospital Nanjing China

**Keywords:** glucolipid metabolism, gut microbiota, perilla oil, type 2 diabetes, ω‐3 polyunsaturated fatty acids

## Abstract

**Scope:**

The aim of this study is to examine whether perilla oil supplementation improves glucolipid metabolism and modulates gut microbiota in diabetic KKAy mice.

**Methods and results:**

The successfully established diabetic KKAy mice are randomized into four groups: diabetic model (DM), low‐dose perilla oil (LPO), middle‐dose perilla oil (MPO), and high‐dose perilla oil (HPO). C57BL/6J mice are fed a chow diet as normal control (NC). At the end of 12 weeks, mice are euthanized and glucolipid indications are analyzed. Gut microbiota analysis is carried out based on the sequencing results on V4 region of 16S rRNA. Although serum glucose, insulin, total cholesterol, low‐density lipoprotein cholesterol, high‐density lipoprotein cholesterol, abundance‐based coverage estimator, and shannon are unchanged, serum triglyceride significantly decreases in LPO compared with DM. The histopathological changes of hepatocellular macrovesicular steatosis and adipocyte hypertrophy are ameliorated by perilla oil supplementation. *Blautia* is significantly decreased in LPO, MPO, and HPO, compared with DM. Nonmetric multidimensional scaling analysis shows NC and LPO are relatively coherent.

**Conclusion:**

These findings indicate that dietary supplementation with perilla oil can improve hypertriglyceridemia and gut dysbiosis in diabetic KKAy mice, which can be associated with potential benefits to human health.

## 1 Introduction

Diabetes is on the rise. According to the first WHO “Global report on diabetes,”^1^ the global prevalence of diabetes has almost doubled since 1980 to 8.5% in the adult population. This dramatic rise poses an immense public health and medical challenge. Moreover, about 80% of patients with diabetes have dyslipidemia,^2^ which might contribute to the development of diabetes.

Diet plays a fundamental role in managing diabetes. In 1976, a survey of the composition of Eskimo food has suggested that the low incidence of diabetes in Greenland Eskimos may partly be explained by the high ω‐3 PUFA content of fish oil, including eicosapentaenoic acid (EPA) and docosahexaenoic acid (DHA).^3^, ^4^ Since then, an increasing number of studies have been carried out to confirm the effects of fish oil‐derived ω‐3 PUFA on glucolipid metabolism in type 2 diabetic patients.^5^, ^6^ Of note, a new study suggests the Inuit population are genetically adapted to consuming a high PUFA diet and therefore the findings from this population may not be fully extendable to other populations.^7^ Our previous study also demonstrated the protective effects of ω‐3 PUFA enriched fish oil against type 2 diabetes.^8^ However, owing to the sustainability and heavy metal pollution of marine sources, the plant‐derived α‐linolenic acid (ALA), which is abundant in perilla oil and flaxseed oil, has become an attractive alternative source to fish oil.^9^, ^10^ Unfortunately, there are still very few studies in‐depth regarding perilla oil intervention research in type 2 diabetes.

In recent years, emerging evidence has revolutionized our understanding of the close relationship between gut microbiota and diabetes.^11^ Qin et al.^12^ identified and validated ≈60 000 type 2 diabetes associated markers by utilizing metagenome‐wide association analysis. Furthermore, gut metagenomes of type 2 diabetic patients have a characteristic distribution of single‐nucleotide polymorphism in *Bacteroides coprocola*.^13^ The Integrative Human Microbiome Project has regarded type 2 diabetes as one of its top priorities to evaluate the interactions of microbiome and host.^14^ Nutrition has an inescapably important role in the gut microbiota homeostasis. The recently published review has primarily focused on gut microbiota changes induced by different macronutrients, such as dietary fat, fiber, and protein.^15^ In December 2016, the International Scientific Association for Probiotics and Prebiotics updated the definition of a prebiotic.^16^ PUFA, for the first time, ranks as one of prebiotic. However, the effect of perilla oil‐derived ω‐3 PUFA supplementation on gut microbiota in type 2 diabetes is still unclear.

Therefore, the aim of this study was to examine whether perilla oil‐derived ω‐3 PUFA would improve glucolipid metabolism and thereby modulate gut microbiota in diabetic KKAy mice.

## 2 Experimental Section

### 2.1 Materials

Perilla oil samples were manufactured by Sinolife United Co., Ltd (Nanjing, China) according to our requirements. The ALA concentration was 60.43% (Table S1, Supporting Information).

### 2.2 Animals and Treatment

Eight‐week‐old spontaneously diabetic male KKAy mice were obtained from Beijing HFK Bioscience Co., Ltd (Beijing, China), and age‐matched male nondiabetic C57BL/6J mice were obtained from Model Animal Research Center of Nanjing University (Nanjing, China). All mice were housed one per cage at 22 ± 2 °C with a 12‐h light/dark cycle. The KKAy mice were fed a high fat diet, which has applied for national invention patent of China (application number: CN201110127312.5). The C57BL/6J mice (*n* = 10) were fed normal chow die as normal control (NC). The composition of the diets is shown in **Table** 1. The fatty acid profile and ingredients of the diets are shown in Table S2–S4, Supporting Information. After 5 weeks of high fat diet feeding when the fasting blood glucose was higher than 13.9 mmol L^–1^, the KKAy mice were randomly divided into four groups (*n* = 10 in each group): 1) diabetic model (DM), 2) low dose perilla oil (LPO), 3) middle dose perilla oil (MPO), and 4) high dose perilla oil (HPO). NC and DM were gavaged with 0.5% sodium carboxy methylcellulose. The animal treatment lasted for 12 weeks, during which both diet and water were consumed ad libitum. At the end of experiment, overnight fasted mice were killed with sodium pentothal. Blood and tissue samples were collected for further analysis. The animal experimental protocols were conducted according to the Institutional Animal Care and Use Committee of Southeast University (no. 2015‐0910‐008).

**Table 1:** Composition of the diets (per 100 g)

	High‐fat diet	Chow diet
Carbohydrate [g]	40.3	72.7
Protein [g]	17.5	12.5
Fat [g]	21.5	4.0
Energy from carbohydrate [%]	37.9	77.2
Energy from protein [%]	16.5	13.3
Energy from fat [%]	45.6	9.5

### 2.3 Dosage Information

According to Chinese Dietary Reference Intakes^17^ and previous study,^8^ the recommended human daily intake of perilla oil was 4 g. As Technical Standards for Testing & Assessment of Health Food in China suggests, there was an equivalent dosage for mice about ten (0.67 g kg^–1^ bw d^–1^ for LPO), 20 (1.33 g kg^–1^ bw d^–1^ for MPO), 30 (2.00 g kg^–1^ bw d^–1^ for HPO) times of that used in human. Perilla oil supplementation was administered by gavage once a day from 2 to 5 pm. The dose is achievable via available supplements.

### 2.4 Serum Glucolipid Metabolism

Serum glucose, triglyceride (TG), total cholesterol (TC), LDL cholesterol (LDL‐C), and HDL cholesterol (HDL‐C) were determined by automatic biochemical analyzer (Beckman, DxC800, USA). Serum insulin was measured using ELISA kits (Nanjing Jiancheng Bioengineering Institute, Nanjing, China) according to the manufacturer's protocol.

### 2.5 Histological Examination

Liver, epididymal white adipose, and interscapular brown adipose tissues from each mouse were immediately fixed with formalin and stained with hematoxylin and eosin staining. The degrees of distribution were used to determine lesion including hepatocellular macrovesicular steatosis (liver tissue), adipocyte hypertrophy (white adipose tissue), and single bubble adipocyte accumulation (brown adipose tissue). The histopathological findings were scored as 0  (absent), 1  (<5%), 2  (5–29%), 3  (30–49%), 4  (50–75%), and 5  (>75%). Three fields were scored for each sample. The viewer was blinded to the sample groups.

### 2.6 Gut Microbiota Analysis

Ten mice were selected from each group for gut microbiota analysis. Bacterial genomic DNA was isolated from mouse colonic feces using the FastDNA SPIN Kit for Feces (MP Biomedicals, USA) according to the manufacturer's instructions. The V4 hypervariable region of the bacterial 16S rRNA gene was amplified using universal primers (515F and 806R). Sequencing was performed on Illumina HiSeq platform by HiSeq2500 PE250 (Illumina, USA). The sequencing depth in each sample is listed in Table S5, Supporting Information. Sequences with ≥97% similarity were assigned to the same operational taxonomic units (OTUs). The alpha diversity was calculated using abundance‐based coverage estimator (ACE),^18^ Shannon and beta diversity was determined using multiple response permutation procedure, nonmetric multidimensional scaling (NMDS), and *t*‐test.

### 2.7 Statistical Analysis

Data are expressed as mean ± SD. Significant differences among treatment factors were analyzed by one‐way analysis of variance followed by Tukey's post hoc test using PASW statistics 18.0 (SPSS Inc, USA). The significance threshold was set at a *p* value of less than 0.05.

## 3 Results

### 3.1 Physical Features

Initial body weight, final body weight, body length, liver weight, perirenal fat, and epididymal fat were significantly increased in DM than that in NC (**Table** 2). Compared with DM, perirenal fat was significantly increased in LPO, MPO, and HPO. There was no significant difference in initial body weight, final body weight, body length, liver weight, and epididymal fat among perilla oil intervention groups.

**Table 2:** Physical features of mice

	NC	DM	LPO	MPO	HPO
Initial body weight [g]	27.4 ± 2.3	41.3 ± 3.4*	41.5 ± 3.8*	41.2 ± 3.0*	39.6 ± 2.7*
Final body weight [g]	29.8 ± 3.3	45.6 ± 3.5*	46.4 ± 3.7*	45.6 ± 3.1*	45.7 ± 2.5*
Body length, [cm]	9.8 ± 0.3	10.2 ± 0.3*	10.2 ± 0.4*	10.1 ± 0.2*	10.0 ± 0.2*
Liver weight [g]	1.127 ± 0.136	5.241 ± 0.590*	4.548 ± 1.147*	4.922 ± 0.714*	4.832 ± 0.850*
Perirenal fat [g]	0.201 ± 0.107	0.625 ± 0.193*	0.889 ± 0.189*,, #	0.889 ± 0.291*,, #	0.916 ± 0.173*,, #
Epididymal fat [g]	0.574 ± 0.089	0.887 ± 0.279*	0.974 ± 0.229*	0.963 ± 0.362*	0.869 ± 0.302*

Data are expressed as mean ± SD. NC, normal control; DM, diabetic model; LPO, low dose perilla oil; MPO, middle dose perilla oil; HPO, high dose perilla oil

^*^ *p* < 0.05 versus NC

^#^ *p* < 0.05 versus DM.

### 3.2 Serum Lipids, Glucose, and Insulin Levels

Serum glucose, insulin, TG, TC, LDL‐C, and HDL‐C were significantly increased in DM than that in NC (**Table** 3). Compared with DM, significantly lower TG was observed in LPO (95% confidence interval: 0.0008–0.8772; Figure S1, Supporting Information). There was no significant difference in glucose, insulin, TC, LDL‐C, and HDL‐C among perilla oil intervention groups.

**Table 3:** Effect of perilla oil supplementation on serum glucolipid metabolism

	NC	DM	LPO	MPO	HPO
Glucose [mmol L^–1^]	6.78 ± 1.01	14.49±1.78*	17.87 ± 1.76*	14.73 ± 3.79*	14.96 ± 3.08*
Insulin [mIU L^–1^]	9.34 ± 1.56	14.26±3.25*	15.23 ± 2.90*	14.42 ± 3.38*	15.61 ± 4.33*
TG [mmol L^–1^]	1.50 ± 0.38	2.19±0.54*	1.77 ± 0.35#	2.02 ± 0.46*	2.12 ± 0.66*
TC [mmol L^–1^]	2.61 ± 0.36	7.07±1.16*	5.92 ± 1.19*	7.22 ± 1.56*	7.04 ± 1.46*
LDL‐C [mmol L^–1^]	0.32 ± 0.04	0.96 ± 0.30*	0.94 ± 0.31*	0.99 ± 0.27*	0.90 ± 0.17*
HDL‐C [mmol L^–1^]	1.76 ± 0.27	4.14 ± 0.38*	4.30 ± 0.48*	4.49 ± 0.69*	4.03 ± 0.80*

Data are expressed as mean ± SD. NC, normal control; DM, diabetic model; LPO, low dose perilla oil; MPO, middle dose perilla oil; HPO, high dose perilla oil; TG, triglyceride; TC, total cholesterol; LDL‐C, low density lipoprotein cholesterol; HDL‐C, high density lipoprotein cholesterol

^*^ *p* < 0.05 versus NC

^#^ *p* < 0.05 versus DM.

### 3.3 Histological Examination

**Figure 1:**
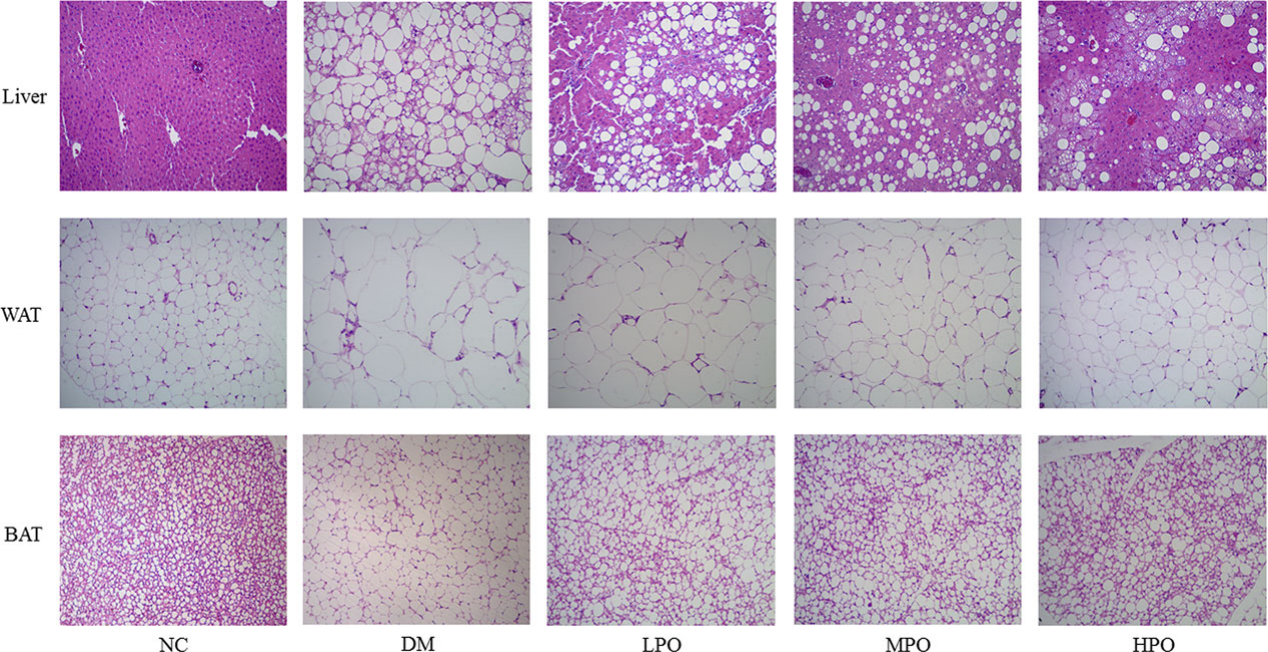
Effect of perilla oil supplementation on histopathological changes of liver, white adipose, and brown adipose tissue. Hematoxylin and eosin staining, ×100 magnification. WAT, white adipose tissue; BAT, brown adipose tissue; NC, normal control; DM, diabetic model; LPO, low dose perilla oil; MPO, middle dose perilla oil; HPO, high dose perilla oil.

Histological examination of liver, white adipose, and brown adipose tissue in NC showed normal cell architecture (**Figure** 1, **Table** 4). However, the histological evaluation in DM revealed serious hepatocellular macrovesicular steatosis, adipocyte hypertrophy, and a large number of single bubble adipocyte. These histopathological changes of liver and white adipose tissue were ameliorated by perilla oil supplementation, especially in HPO.

**Table 4:** Effect of perilla oil supplementation on histological changes

	NC	DM	LPO	MPO	HPO
Liver	–	4.78 ± 0.44	4.44 ± 0.73	3.82 ± 1.25*	3.60 ± 1.17*
White adipose tissue	–	4.30 ± 0.82	4.10 ± 0.88	3.91 ± 1.14	3.00 ± 0.87*
Brown adipose tissue	–	4.30 ± 0.95	4.10 ± 1.20	3.64 ± 1.29	3.60 ± 1.17

Data are expressed as mean ±SD. NC, normal control; DM, diabetic model; LPO, low dose perilla oil; MPO, middle dose perilla oil; HPO, high dose perilla oil

^*^ *p* < 0.05 versus DM.

### 3.4 Overall Structural Changes of Gut Microbiota

**Figure 2:**
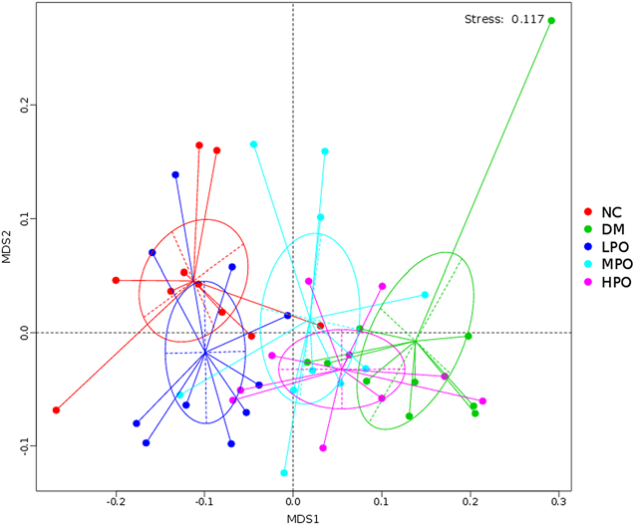
Nonmetric multidimensional scaling ordination. MDS, multidimensional scaling; NC, normal control; DM, diabetic model; LPO, low dose perilla oil; MPO, middle dose perilla oil; HPO, high dose perilla oil.

In all detected OTUs, 568 were shared by all groups (Figure S2, Supporting Information). The unique OTUs were 25, 49, 18, 7, and 26 in NC, DM, LPO, MPO, and HPO, respectively. There was no significant difference in the value of ACE and shannon among the five groups (Figure S3, Supporting Information). Significant difference of beta‐diversity distance was found between NC, LPO, and DM, NC and LPO groups were more similar than NC to the other groups (**Table** 5). NMDS analysis showed NC and LPO were relatively coherent (**Figure** 2).

**Table 5:** The beta‐diversity distances between groups

	Observed Δ	Expected Δ	*p*‐Value
NC‐DM	0.5797	0.6718	0.001
NC‐LPO	0.5681	0.6321	0.001
NC‐MPO	0.5913	0.6455	0.001
NC‐HPO	0.5756	0.6584	0.001
DM‐LPO	0.6449	0.6788	0.004
DM‐MPO	0.6681	0.6714	0.254
DM‐HPO	0.6524	0.6475	0.654
LPO‐MPO	0.6565	0.6561	0.458
LPO‐HPO	0.6407	0.6571	0.051
MPO‐HPO	0.664	0.6579	0.825

### 3.5 Key Phylotypes in Response to Perilla Oil Supplementation

**Figure 3:**
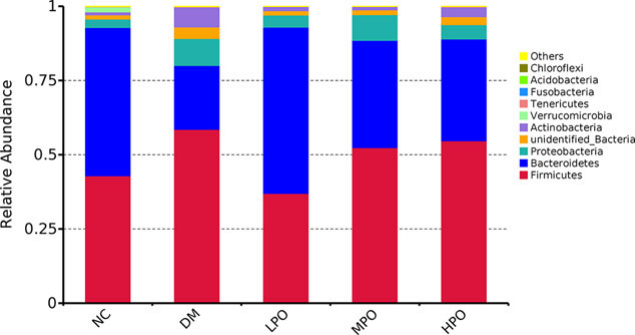
The relative abundance of gut microbiota at phylum level. NC, normal control; DM, diabetic model; LPO, low dose perilla oil; MPO, middle dose perilla oil; HPO, high dose perilla oil.

**Figure 4:**
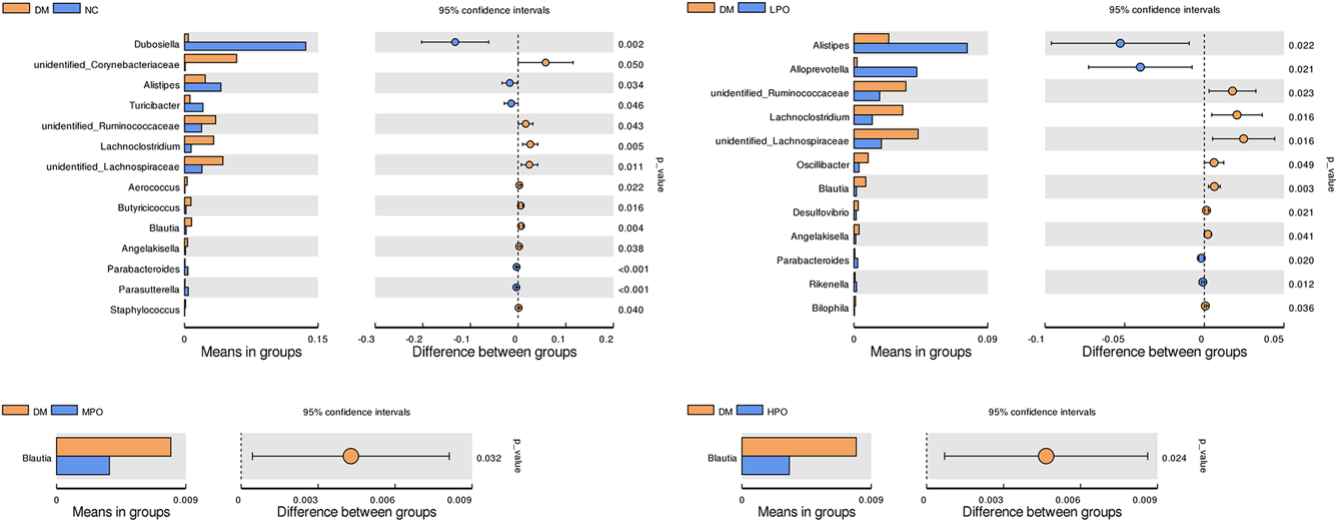
Comparison of gut microbiota at genus level. NC, normal control; DM, diabetic model; LPO, low dose perilla oil; MPO, middle dose perilla oil; HPO, high dose perilla oil.

**Figure 5:**
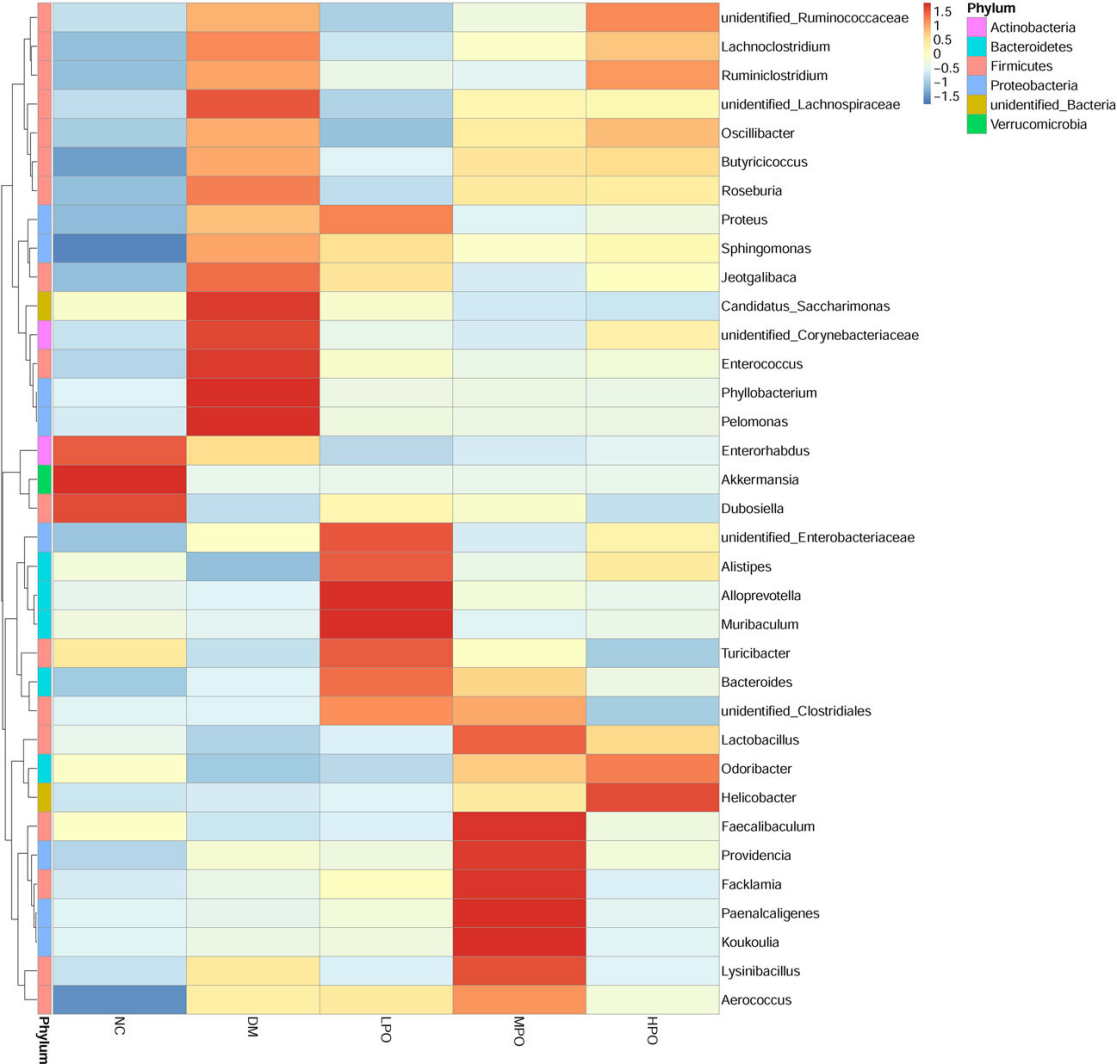
Hierarchically clustered heat map analysis of the top 35 abundance of gut microbe at genus level. NC, normal control; DM, diabetic model; LPO, low dose perilla oil; MPO, middle dose perilla oil; HPO, high dose perilla oil.

At phyla level, Firmicutes, Bacteroidetes and Actinobacteria were the dominant phyla in all groups (**Figure** 3). At genus level, *unidentified_Corynebacteriaceae*, *unidentified_Ruminococcaceae*, *Lachnoclostridium*, *unidentified_Lachnospiraceae*, *Aerococcus*, *Butyricicoccus*, *Blautia*, *Angelakisella*, and *Staphylococcus* significantly increased and *Dubosiella*, *Alistipes*, *Turicibacter*, *Parabacteroides*, and *Parasutterella* significantly decreased in DM relative to NC (**Figure** 4). Compared with DM, *Alistipes*, *Alloprevotella*, *Parabacteroides*, and *Rikenella* significantly increased, *unidentified_Ruminococcaceae*, *Lachnoclostridium unidentified_Lachnospiraceae*, *Oscillibacter*, *Blautia*, *Desulfovibrio*, *Angelakisella*, and *Bilophila* significantly decreased in LPO, and *Blautia* significantly decreased in MPO and HPO. Ternaryplot analysis showed *Dubosiella* and *Turicibacter* were the most abundant genus in LPO relative to MPO and HPO, *Lactobacillus* was the most abundant genus in HPO relative to LPO and MPO (Figure S4, Supporting Information). The heatmap analysis of the top 35 genus is displayed in the **Figure** 5. *Akkermansia* was high in NC and low in DM. The percentage of *Akkermansia* was unaffected by supplementation of perilla oil.

## 4 Discussion

According to the latest results from the Diabetes UK‐James Lind Alliance Priority Setting Partnership, the issue concerning what role do fats have in the management of type 2 diabetes became one of top ten research priorities for type 2 diabetes.^19^ In this study, we determine whether perilla oil supplementation improves glucolipid metabolism and modulates gut microbiota in diabetic KKAy mice. While serum glucose, insulin, TC, LDL‐C, and HDL‐C were not significantly altered by perilla oil supplementation, our analysis did detect a significant TG‐lowering effect in LPO. In addition, the histopathological changes of hepatocellular macrovesicular steatosis and adipocyte hypertrophy in epididymal fat were ameliorated by perilla oil supplementation. Furthermore, perilla oil supplementation improved the gut dysbiosis by decreasing the abundance of *Blautia*.

Perilla oil is an edible plant oil extracted from perilla seeds, which is both food and herbal medicine in China. Perilla oil is rich in ALA, a plant‐derived essential ω‐3 PUFA. Although perilla oil‐derived ALA is one of ω‐3 PUFA, its antidiabetic effect is little‐studied relative to fish oil‐derived ω‐3 PUFA. The KKAy mouse, also named yellow KK mouse, carries the yellow obese and diabetes genes. This animal model is characterized by marked obesity, hyperinsulinemia, hyperglycemia, and dyslipidemia.^20^ As expected, serum glucolipid metabolism and pathological changes in liver and adipose tissue deteriorated in DM. After 12 weeks of perilla oil supplementation, serum TG was decreased. Furthermore, the observed decrease in serum TG following perilla oil consumption was associated with favorable histopathological changes in liver and white adipose tissue. Likewise, Iizuka et al.^21^ reported that a combination of fish oil‐derived ω‐3 PUFA and pioglitazone positively changes adipocytes in KK mice. In ob/ob mouse, an animal model of insulin resistance and type 2 diabetes, MUFA‐rich diets with ALA improved both glycemic responses and insulin sensitivity.^22^ MUFA‐rich diets with EPA and/or DHA had significantly lower TG, but glucose and insulin sensitivity were not improved. Pachikian et al.^23^ suggested that the lack of dietary ω‐3 PUFA can be part of hepatotoxic events linked to steatosis. Our previous study has shown that only once oral high fat meal can increase serum TG in abdominal obesity patients with high postprandial insulin resistance^24^ and type 2 diabetic patients with abdominal obesity.^25^ Moreover, plant‐derived oil rich in ω‐3 PUFA was able to improve lipid metabolism in Sprague–Dawley rats fed a high fat diet.^26^ Yang et al.^27^ found that an increase in ω‐3 PUFA levels and the concomitant decrease in the ω‐6/ω‐3 PUFA level ratio are likely to be involved in the beneficial changes to the metabolic indicators. In contrast, serum TG was unaltered by consumption of flaxseed/fish oil mixture for 16 weeks in C57BL/6J mice.^28^ This inconsistency may be partly explained by differences in species, oil types, and duration. Of note, PUFA is highly susceptible to peroxidation. To date, the effects of oxidized oil rich in ALA on glucolipid biomarkers are inconclusive. In LDL‐receptor knockout mice, glucose levels and lipid profile did not differ between fresh flaxseed oil and heated flaxseed oil.^29^ Whether oxidized perilla oil has a specific effect on glucolipid metabolism still needs to be elucidated.

Interestingly, we observe greater perirenal fat pads in animals receiving the perilla oil gavage. However, epididymal fat weights are similar in LPO, MPO, HPO, and DM. This phenomenon was probably caused by the higher dosage in the background of high fat diet, because perilla oil was also the major source of energy production. In addition, there are regional differences in preadipocyte adipogenesis. Tchkonia et al.^30^ contend that single human or rodent preadipocytes derived from different depots and cultured under identical conditions retain distinct capacities for adipogenesis, despite originating from the same individuals. Kirkland et al.^31^ reported that rat perirenal preadipocytes were capable of more extensive replication than epididymal preadipocytes. Furthermore, fat tissue lipolysis varies among depots. Lu et al.^32^ observed that epididymal fat had stronger lipolytic effect than perirenal fat. The effect of perrilla oil supplementation on the fat deposition linked to different body parts needs to be studied further. This is also a potential health concern worth noting.

Currently, the development of gut microbiota is an emerging area of diabetes research. Impairment of gut microbiota homeostasis plays an essential role in the onset and progression of type 2 diabetes. *Blautia* is a Gram‐positive, anaerobe bacterium belonging to the family *Lachnospiraceae*, which was thought to take part in the development of glucose metabolism disturbances.^33^ In addition, a safflower oil based high‐fat/high‐sucrose diet resulted in an increased abundance of *Blautia*.^34^ In the current study, *Blautia* was much higher in DM compared with NC, but in the gut of low, middle, and high dose perilla oil‐fed KKAy mice, *Blautia* was lower than DM. This finding suggest that *Blautia* may be involved in the improved gut function. Further animal experiments and human studies will be necessary to determine the specific effects of perilla oil on *Blautia* in diabetes.

When compared with LPO and MPO, *Lactobacillus* was the most abundant genus in HPO. *Lactobacillus* is generally believed to be a beneficial bacteria.^35^ It can convert sugars to lactic acid. In a previous publication, Pachikian et al. demonstrated that C57BL/6J mice fed with ω‐3 PUFA depleted diet for two generations exhibited a decrease in *Lactobacillus* in the cecal content as compared to control mice.^36^ In line with this finding, a comparison of lard oil and fish oil revealed that *Lactobacillus* was increased in fish oil fed mice.^37^ Moreover, supplementation of the high fat diet with a combination of ω‐3 PUFA (EPA and DHA) increased the quantities of *Lactobacillus* in diet‐induced obese mice.^38^

Perilla oil gavage may be affecting the gut microbiota via increased bile secretion. Bile acids are the major effectors of digestion and absorption of fat. After the oil gavage, bile acids are released to solubilize fat and processed by the microbiome, giving rise to secondary metabolites, such as SCFAs, trimethylamine, and secondary bile acids.^39^ These secondary metabolites are key for the fat–bile–gut connection. In addition, ALA can exert antimicrobial effect by inhibition of fatty acid synthesis, which is crucial for bacterial survival and growth.^40^ Exact mechanism underlying these effects is worth further study.

Our study had several limitations. First, this study lacks positive control and negative control group, so it is difficult to discern the effect of perilla oil on lessening diabetic conditions more precisely. Second, one time point testing failed to monitor the kinetics of gut microbiota. Finally, care must be taken in extrapolating results to human due to the small sample size and the inherent physiological differences between mouse and human. Further studies are warranted to examine whether the metabolic health outcomes are causally related to changes in intestinal flora.

In conclusion, perilla oil supplementation are not only central to hypertriglyceridemia, but are linked with improvement in gut microbiota. It is recommended that a follow‐up study with a large sample size should be confirmed in clinical trials.

## Conflict of Interest

The authors declare no conflict of interest.

## Supporting information

Table S1. The fatty acid profile of perilla oil.
Table S2. The fatty acid profile of the diets.
Table S3. The ingredients of high fat diet (per 100g).
Table S4. The ingredients of chow diet (per 100g).
Table S5. The sequencing depth in each sample.
Figure S1. Effect of perilla oil supplementation on serum triglyceride. NC, normal control; DM, diabetic model; LPO, low dose perilla oil; MPO, middle dose perilla oil; HPO, high dose perilla oil; TG, triglyceride.^*^ *P* < 0.05 vs. NC, ^#^ *P* < 0.05 vs. DM.
Figure S2. Venn diagram of operational taxonomic units abundance. NC, normal control; DM, diabetic model; LPO, low dose perilla oil; MPO, middle dose perilla oil; HPO, high dose perilla oil.
Figure S3. Comparison of bacterial richness and diversity. ACE, Abundance‐based coverage estimator; NC, normal control; DM, diabetic model; LPO, low dose perilla oil; MPO, middle dose perilla oil; HPO, high dose perilla oil.
Figure S4. Ternaryplot displaying the genus with significantly different abundance. NC, normal control; DM, diabetic model; LPO, low dose perilla oil; MPO, middle dose perilla oil; HPO, high dose perilla oil.
