# Supplementary material for: Perilla Oil Supplementation Improves Hypertriglyceridemia and Gut Dysbiosis in Diabetic KKAy Mice
Source: Mol Nutr Food Res. 2018 Nov 8;62(24):1800299. doi: 10.1002/mnfr.201800299 (PMC6646911; doi:10.1002/mnfr.201800299)
Supplement: Supplementary file 1 — Table S1. The fatty acid profile of perilla oil. Table S2. The fatty acid profile of the diets. Table S3. The ingredients of high fat diet (per 100g). Table S4. The ingredients of chow diet (per 100g). Table S5. The sequencing depth in each sample. Figure S1. Effect of perilla oil supplementation on serum triglyceride. NC, normal control; DM, diabetic model; LPO, low dose perilla oil; MPO, middle dose perilla oil; HPO, high dose perilla oil; TG, triglyceride.* P < 0.05 vs. NC, # P < 0.05 vs. DM. Figure S2. Venn diagram of operational taxonomic units abundance. NC, normal control; DM, diabetic model; LPO, low dose perilla oil; MPO, middle dose perilla oil; HPO, high dose perilla oil. Figure S3. Comparison of bacterial richness and diversity. ACE, Abundance‐based coverage estimator; NC, normal control; DM, diabetic model; LPO, low dose perilla oil; MPO, middle dose perilla oil; HPO, high dose perilla oil. Figure S4. Ternaryplot displaying the genus with significantly different abundance. NC, normal control; DM, diabetic model; LPO, low dose perilla oil; MPO, middle dose perilla oil; HPO, high dose perilla oil. [file MNFR-62-na-s001.pdf]

Table S1. The fatty acid profile of perilla oil

	Percentage (%)
Palmitic acid	5.90
Stearic acid	2.72
Oleic acid	14.59
Linoleic acid	13.43
α-linolenic acid	60.43

Only fatty acids detected in ≥ 1% of total fatty acids are shown.

Table S2. The fatty acid profile of the diets

	High fat diet	Chow diet
Palmitic acid, %	24.3	11.1
Stearic acid, %	13.2	3.8
Palmitoleic acid, %	2.6	1.5
Elaidic acid, %	41.5	22.4
Linoleic acid, %	14.8	51.7
α-linolenic acid, %	1.0	6.7

Only fatty acids detected in ≥ 1% of total fatty acids are shown.

Table S3. The ingredients of high fat diet (per 100g)

	Weight (g)
Corn flour	31.6
Soybean dregs	17.5
Lard oil	11.0
Sucrose	10.0
Yolk powder	10.0
Wheat flour	7.0
Bran	4.0
Mineral mix	2.8
Fish flour	2.5
Wheat germ	1.5
Yeast	1.4
Salt	0.4
Vitamin mix	0.3

Table S4. The ingredients of chow diet (per 100g)

	Weight (g)
Cornstarch	46.6
Dextrinized cornstarch	15.5
Casein	14.0
Sucrose	10.0
Soybean oil	4.0
Fiber	5.0
Mineral mix	3.5
Vitamin mix	1.0
Choline bitartrate	0.2
L-Cystine	0.2

Table S5. The sequencing depth in each sample

Sample name	Raw tags	Clean tags	Effective tags
NC1	65563	64506	63995
NC2	56350	55479	54957
NC3	63678	62853	62067
NC4	67637	46786	39638
NC5	62541	43797	34323
NC6	66942	48994	40387
NC7	72080	51220	44604
NC8	73920	53432	44648
NC9	66530	49643	36055
NC10	71591	50110	31552
DM1	64241	63367	62726
DM2	61234	60434	59635
DM3	66745	65715	65038
DM4	67973	51034	42901
DM5	78496	62165	44612
DM6	68731	54124	39416
DM7	76961	59958	46745
DM8	82743	67136	57493
DM9	61125	60320	59715
DM10	69928	54937	52324

LPO1	62032	61095	60456
LPO2	74280	53620	49164
LPO3	52635	51862	51437
LPO4	67027	50590	33617
LPO5	76945	56564	48256
LPO6	73916	54862	43876
LPO7	70800	51928	48705
LPO8	74558	57025	46728
LPO9	71818	53490	41317
LPO10	75735	65604	61512
MPO1	59618	58703	58050
MPO2	65106	64042	63438
MPO3	56228	55429	54975
MPO4	71723	54954	43610
MPO5	74621	52793	44042
MPO6	80143	56755	46520
MPO7	69691	53530	41172
MPO8	77934	60188	48681
MPO9	73861	57341	49794
MPO10	76746	59776	47607
HPO1	57851	57016	56428
HPO2	55252	54444	53564

HPO3	51582	50903	50408
HPO4	75876	60618	46967
HPO5	80845	65135	54302
HPO6	65891	49765	41023
HPO7	65769	49330	33708
HPO8	81201	66721	56123
HPO9	89728	78360	70404
HPO10	81889	66133	54829

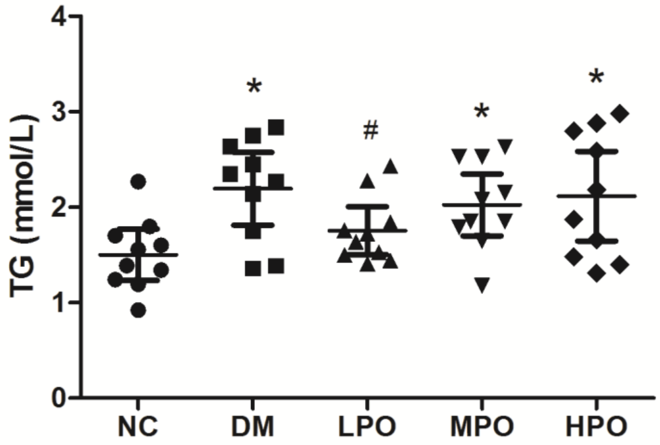

Figure S1. Effect of perilla oil supplementation on serum triglyceride. NC, normal control; DM, diabetic model; LPO, low dose perilla oil; MPO, middle dose perilla oil; HPO, high dose perilla oil; TG, triglyceride. * $P < 0.05$ vs. NC, # $P < 0.05$ vs. DM.

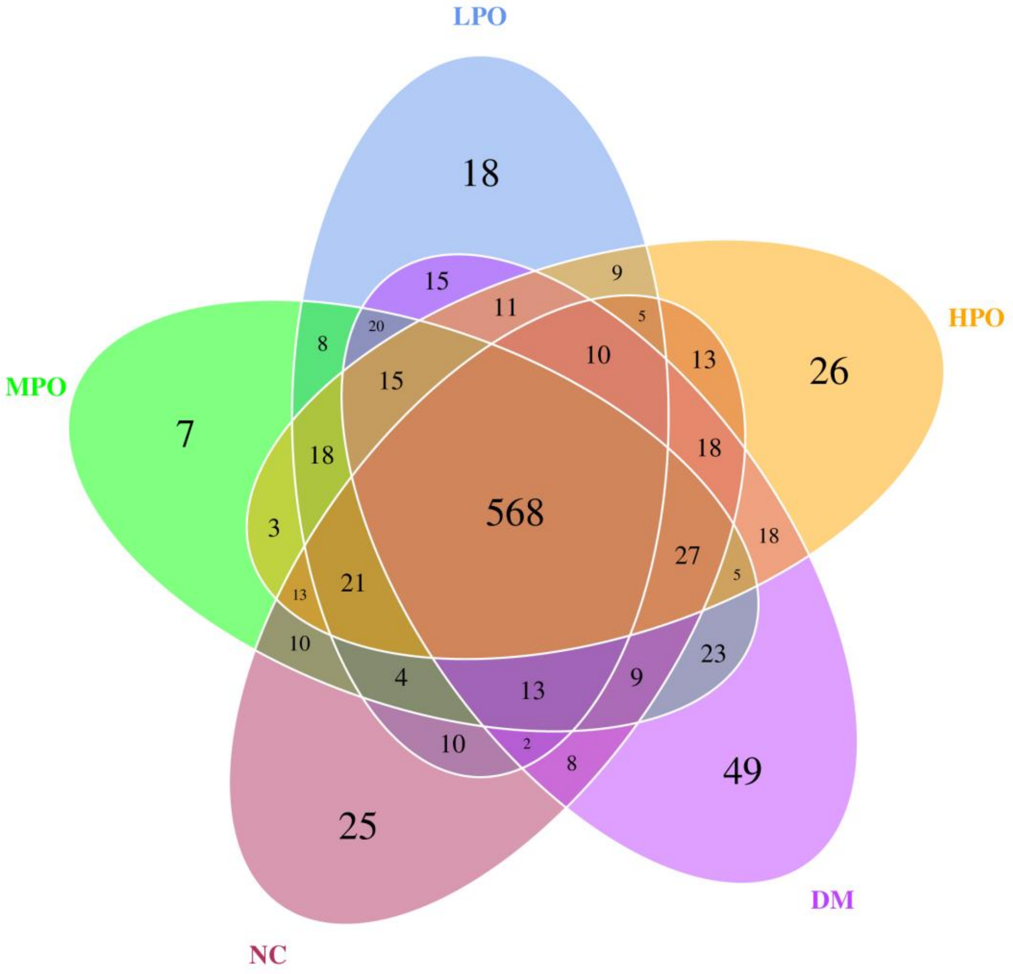

Figure S2. Venn diagram of operational taxonomic units abundance. NC, normal control; DM, diabetic model; LPO, low dose perilla oil; MPO, middle dose perilla oil; HPO, high dose perilla oil.

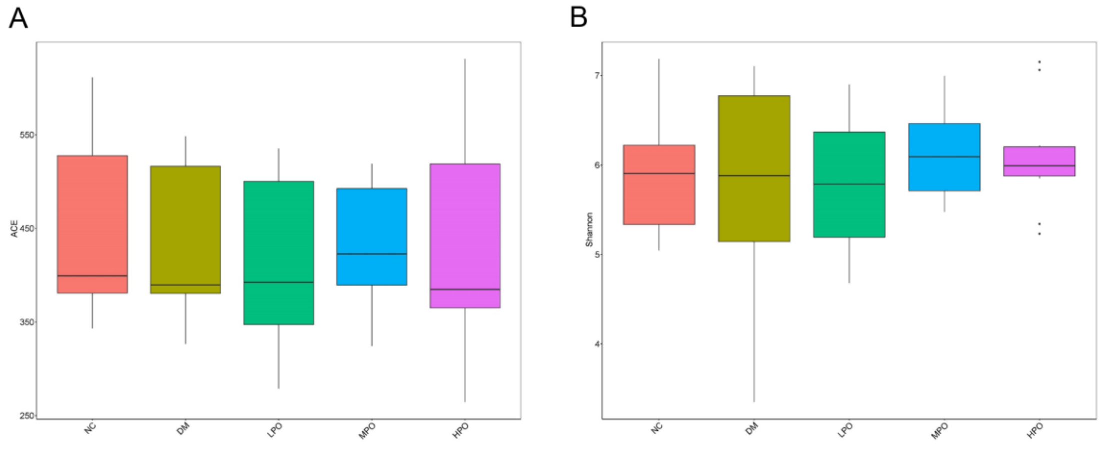

Figure S3. Comparison of bacterial richness and diversity. ACE, Abundance-based coverage estimator; NC, normal control; DM, diabetic model; LPO, low dose perilla oil; MPO, middle dose perilla oil; HPO, high dose perilla oil.

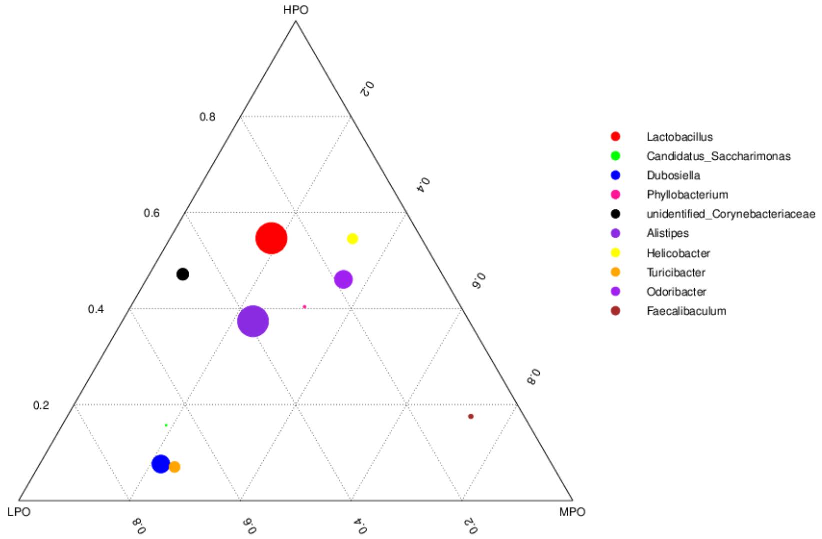

Figure S4. Ternaryplot displaying the genus with significantly different abundance. NC, normal control; DM, diabetic model; LPO, low dose perilla oil; MPO, middle dose perilla oil; HPO, high dose perilla oil.
